# High Temperature Deformation of Twin-Roll Cast Al-Mn-Based Alloys after Equal Channel Angular Pressing

**DOI:** 10.3390/ma8115401

**Published:** 2015-11-12

**Authors:** Přemysl Málek, Michaela Šlapáková Poková, Miroslav Cieslar

**Affiliations:** Department of Physics of Materials, Charles University in Prague, Faculty of Mathematics and Physics, Ke Karlovu 5, Prague 2 12116, Czech Republic; malek@met.mff.cuni.cz (P.M.); cieslar@met.mff.cuni.cz (M.C.)

**Keywords:** Al-Mn-based alloys, TRC, ECAP, microstructure stability, high temperature mechanical properties

## Abstract

Twin roll cast Al-Mn- and Al-Mn-Zr-based alloys were subjected to four passes of equal channel angular pressing. The resulting grain size of 400 nm contributes to a significant strengthening at room temperature. This microstructure is not fully stable at elevated temperatures and recrystallization and vast grain growth occur at temperatures between 350 and 450 °C. The onset of these microstructure changes depends on chemical and phase composition. Better stability is observed in the Al-Mn-Zr-based alloy. High temperature tensile tests reveal that equal channel angular pressing results in a softening of all studied materials at high temperatures. This can be explained by an active role of grain boundaries in the deformation process. The maximum values of ductility and strain rate sensitivity parameter *m* found in the Al-Mn-Zr-based alloy are below the bottom limit of superplasticity (155%, *m* = 0.25). However, some features typical for superplastic behavior were observed—the strain rate dependence of the parameter *m*, the strengthening with increasing grain size, and the fracture by diffuse necking. Grain boundary sliding is believed to contribute partially to the overall strain in specimens where the grain size remained in the microcrystalline range.

## 1. Introduction

Aluminum-manganese alloys are frequently used in industrial applications. Their mechanical properties are affected by addition of Fe and Si, which form together with Mn coarse primary particles during solidification or secondary particles during thermal treatment [1,2]. Recovery and recrystallization processes which determine the high temperature mechanical properties can be significantly influenced by the addition of small amounts of Zr. Finely dispersed coherent Al_3_Zr particles formed by suitable thermal treatment pin both dislocations and grain boundaries, retard the course of restoration processes, and enhance the thermal stability of these materials [3,4].

The method of twin-roll casting (TRC) is an alternative to conventional casting techniques of aluminum alloys [5]. Besides the economic benefits, TRC results in microstructure and phase composition modifications—finer grain size, higher solid solution supersaturation, and finer dispersion of particles which can improve mechanical properties of these materials [6]. The benefits of TRC were verified among others on Al-Mn-based alloys [7,8].

The grain size *d* is one of the principal tools for controlling mechanical properties of polycrystalline materials because the reduction in grain size results generally in a strength increase at lower temperatures and a formability enhancement at elevated temperatures. Severe plastic deformation (SPD) of bulk billets achieves the ultra-fine-grained structure by imposing a very high plastic strain [9]. The most common SPD technique is equal channel angular pressing (ECAP) where the sample is pressed through a die consisting of two channels of equal cross-section that intersect at an angle usually 90° [10]. The main advantage of this technique is the possibility of repeating the pressing several times to induce a required level of strain into the material without significant shape changes of pressed samples.

The combination of TRC and ECAP is relatively scarce in the literature. Our previous research revealed that this processing route resulted in a significant microstructure refinement in the Al-Mn-based alloys [11,12]. The formation of individual submicrometer grains along the boundaries of elongated grains of the TRC material occurred already during the first ECAP pass. Four ECAP passes were sufficient for the formation of submicrocrystalline microstructure (grain size of about 400 nm). Nevertheless, not all boundaries had a high-angle character and numerous regions of the size about 5 μm composed of submicrometer subgrains were still present. Further increase in the number of ECAP passes did not result in a further microstructure refinement [13]. The material after four ECAP passes was therefore used for further experiments.

The grain refinement during ECAP causes an increase in strength characteristics at room temperature [14]. On the other hand, the high strain rate superplasticity is frequently observed at temperatures between 400 and 500 °C in Al-alloys with a similar microstructure [15,16]. Such behaviour is conditioned by the stability of the fine-grained microstructure. The experiments performed on the ECAP Al-Mn-based alloys revealed that, in this temperature range, recrystallization and grain growth occurred [11,17]. These processes can be influenced by modification of chemical and phase composition or by unconventional treatment.

Some special applications of Al-Mn alloys connected with deep drawing of thicker blanks require good and uniform formability of the material approaching the superplastic flow. Such behavior was found in an ingot cast Al-Mn-Sc-Zr alloy after SPD [18]. A small amount of Sc was added into the alloy in order to form a fine dispersion of Al_3_Sc and Al_3_(Sc,Zr) precipitates. These precipitates enable the fine-grained structure to hold even at deformation temperatures above 400 °C, which is the necessary prerequisite for superplastic deformation. However, high costs of the Sc-containing alloy limit the industrial use of the material. Therefore a billet from modified Al-Mn-based AA3003-type alloy with a small addition of Zr was prepared by unconventional processing combining high supersaturation and fine primary particle size due to TRC and ultra-fine-grained structure by ECAP.

The main aim of the present research is to investigate high temperature plastic deformation of two TRC Al-Mn-based alloys after grain refinement through four ECAP passes and to verify the possibility of their superplastic behavior. The microstructure investigation using transmission and scanning electron microscopy was performed in order to explain the results of mechanical tests.

## 2. Materials and Methods

Two aluminum alloys from the AA3003 series were studied. The first one has nominal composition 1.0–1.5 wt.% Mn, ≤0.7 wt.% Fe, ≤0.6 wt.% Si, 0.05–0.2 wt.% Cu, and ≤0.2 wt.% Zn (marked as alloy “AlMn”). The second one was modified by an addition of 0.16 wt.% Zr (marked as alloy “AlMnZr”) in order to improve the high temperature stability of the material through the pinning effect of Al_3_Zr particles. Both alloys were prepared by twin-roll casting in industrial conditions to thickness 8.5 mm and width 1 m. The initial TRC materials have elongated grains parallel to the rolling direction with the size in the order of 100 µm in the rolling direction and 50 µm in the perpendicular one [19]. Primary particles of the α-Al(Mn,Fe)Si phase [20,21] with an average size of 1 µm were detected. After casting, parts of both materials were annealed at 450 °C for 8 h in an air furnace (the heating rate 0.5 K/min and water quenching) [22]. This annealing led to the formation of secondary α-Al(Mn,Fe)Si particles with an average size of 100 nm and to the precipitation of coherent particles of the metastable Al_3_Zr phase with a diameter of about 10 nm [22]. Simultaneously, the matrix phase was depleted of Mn atoms [17].

Both non-annealed (marked as “NA”) and annealed (marked as “A”) materials were subjected to 4 passes of equal channel angular pressing at room temperature with rotation B_c_, where material is rotated by 90° after each pass through the ECAP device [10]. The pressing speed was 10 mm/min.

The influence of structure changes occurring during isochronal annealing (50 K/50 min) at temperatures up to 600 °C was monitored by Vickers microhardness measurement performed at room temperature (with the load of 100 g). At least 10 measurements were performed for each state with a standard deviation of approximately 2%.

The high temperature mechanical properties were tested on rectangular tensile samples cut parallel to the pressing direction with the gauge length of 17 mm and cross section typically of 1 × 6 mm^2^ in the temperature range between 350 and 450 °C. A special tensile test was used. After straining to 10% of elongation at the strain rate of 10^−3^ s^−1^ the strain rate was reduced to 4 × 10^−5^ s^−1^ and then gradually increased (after achieving the steady state stress) in steps up to 3 × 10^−2^ s^−1^. The initial parts of deformation curves (up to 10% of elongation) exhibited nearly steady state character which made it possible to evaluate not only the yield stress (*YS*) but also the ultimate tensile strength (*UTS*) without any influence of following strain rate changes. The rest parts of curves were used for the evaluation of the strain rate sensitivity parameter *m* which is a good indicator of possible superplastic behavior. The parameter *m* was evaluated from individual steps as log(σ_2_/σ_1_)/log(${\dot{\varepsilon }}_{2}/{\dot{\varepsilon }}_{1}$) where σ_1_ and σ_2_ are the steady stress values corresponding to strain rates ${\dot{\varepsilon }}_{1}$ and ${\dot{\varepsilon }}_{2}$, respectively. The main advantage of such experiments is that the dependence of the parameter *m* on strain rate in a broad range can be obtained from measurements on one sample. The main disadvantage is that the measured ductility does not correspond to a definite strain rate which can be important in materials exhibiting a strong dependence of deformation characteristics on the strain rate. Therefore the tensile tests at a constant applied strain rate were additionally carried out under conditions corresponding to the maximum strain rate sensitivity.

The microstructure of all materials was studied using scanning electron microscopy equipped with electron back-scatter diffraction (EBSD) in order to reveal the influence of annealing. The additional influence of straining on microstructure was studied using EBSD and transmission electron microscopy (TEM). Both the non-strained material from the grip region of tensile samples (shows the influence of pure annealing) and the strained material from the gauge length close to the fracture point (shows the additional influence of straining) were investigated.

## 3. Results

**Figure 1:**
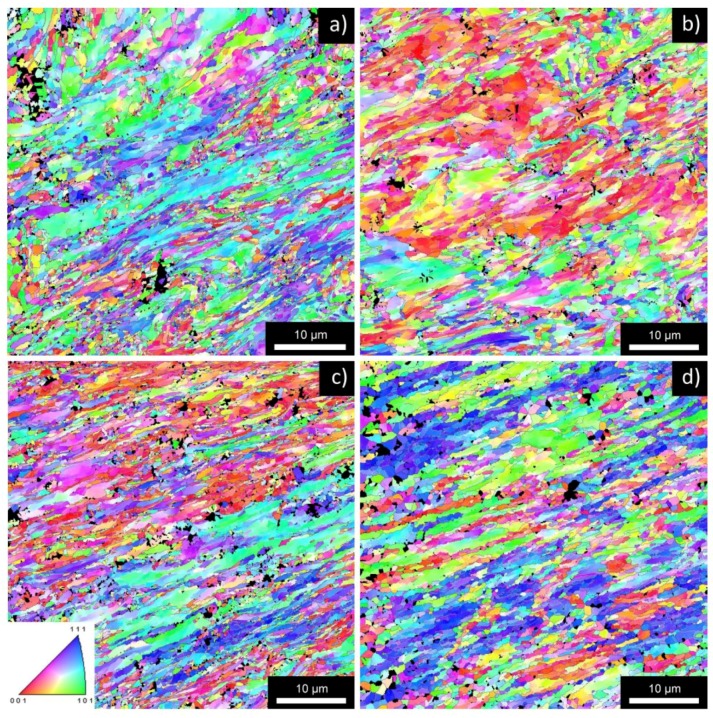
EBSD inverse pole figures of typical areas of the AlMn alloy in the NA state (**a**) and A state (**b**); and of the AlMnZr alloy in the NA state (**c**) and A state (**d**); all materials after four ECAP passes.

The microstructure after four ECAP passes is documented in Figure 1. The microstructure of all materials is dramatically refined due to ECAP and contains a mixture of elongated grains containing low-angle boundaries and small equi-axed grains with the average size of about 400 nm [19]. The fraction of high-angle boundaries computed from the total length of boundaries depends significantly on the accepted threshold value of the misorientation angle. When misorientations exceeding 2° are included into the evaluation procedure the fraction of high-angle boundaries is close to 40%. In the case that only misorientations exceeding 5° are considered, the fraction of high-angle boundaries is close to 60%. In any case, all studied materials contain a large number of low-angle boundaries after four passes of ECAP.

**Figure 2:**
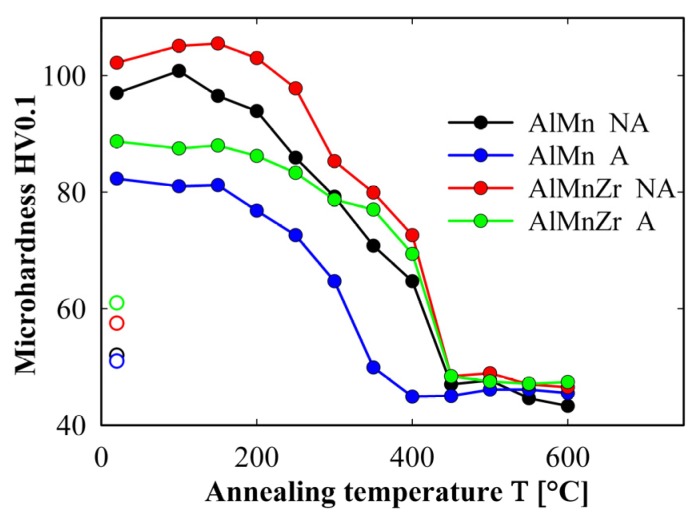
The influence of annealing temperature on the room temperature microhardness for materials after four ECAP passes, open symbols correspond to materials prior to ECAP.

The influence of isochronal annealing on room temperature microhardness is shown in Figure 2 for all materials after four ECAP passes. The data plotted for 20 °C correspond to states after four ECAP passes, *i.e.*, prior to isochronal annealing. For comparison the HV data of all TRC materials prior to ECAP (open symbols in Figure 2) are also plotted. An important strengthening of all materials at 20 °C due to ECAP (compare full and open symbols for 20 °C) can be seen. Higher strengthening due to ECAP occurred in the non-annealed materials. The strengthening effect of Zr addition is also clearly documented. The hardness curves show that softening starts already at about 150 °C and can be divided into two stages. In the first stage a slower decrease of HV with increasing temperature is observed. The final steep drop of HV occurs between 300 and 350 °C in the annealed AlMn alloy and between 400 and 450 °C in all other materials.

**Figure 3:**
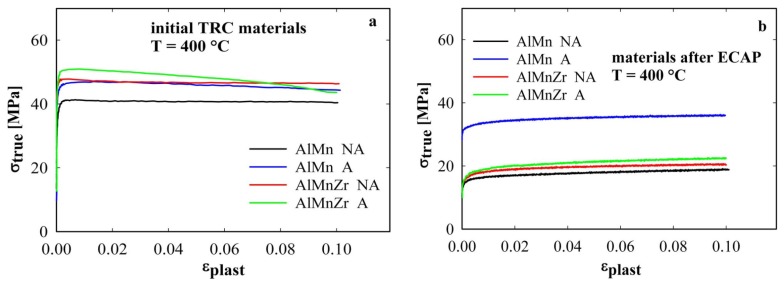
Initial parts of deformation curves at 400 °C of initial TRC materials (**a**) and materials after four ECAP passes (**b**).

The hardness curves of HV in Figure 2 suggest that important microstructure changes occur in the temperature range close to 400 °C. Therefore, the high temperature tensile tests were performed at this temperature in all studied materials. The main deformation characteristics of all ECAP materials (yield stress (*YS*), ultimate tensile strength (*UTS*), ductility (*A*) and maximum value of the strain rate sensitivity parameter (*m_max_*)) evaluated from tensile tests performed at straining temperature 400 °C are summarized in Table 1 and are compared with those of initial TRC materials prior to ECAP. The initial parts of deformation curves (up to 10% of elongation where the applied strain rate was constant 10^−3^ s^−1^) measured at 400 °C are given in Figure 3 both for initial TRC materials prior to ECAP (a) and for materials after four ECAP passes (b). The initial TRC materials do not exhibit any marked strain hardening (Figure 3a), the slight softening observed in AlMnZr materials can be explained by the non-homogeneous deformation starting already at very low strains. The ductility of initial materials prior to ECAP was found to vary from 12% for the annealed AlMnZr alloy to 28% in the non-annealed AlMnZr alloy. The strength at 400 °C of all materials after ECAP is significantly lower in comparison with initial TRC materials prior to ECAP (Figure 3b). The highest value was found in the annealed AlMn alloy, all other materials behave very similarly. The lowest ductility of 22% was observed in the annealed AlMn alloy, the highest one of 119% was observed in the non-annealed AlMnZr alloy (see Table 1).

**Table 1:** Deformation characteristics at 400 °C of all materials in the initial TRC state and after four ECAP passes—yield stress (*YS*), ultimate tensile strength (*UTS*), ductility (*A*) and maximum value of the strain rate sensitivity parameter (*m_max_*).

Material	*YS* [MPa]	*UTS* [MPa]	*A* [%]	*m_max_*
AlMn NA initial TRC	40.5	40.9	21	0.07
AlMn NA ECAP	15.3	17.2	113	0.21
AlMn A initial TRC	45.7	46.7	22	0.06
AlMn A ECAP	32.1	33.9	22	0.04
AlMnZr NA initial TRC	47.9	47.9	28	0.06
AlMnZr NA ECAP	16.4	19.0	119	0.25
AlMnZr A initial TRC	50.4	50.8	12	0.06
AlMnZr A ECAP	16.8	20.6	61	0.20

**Figure 4:**
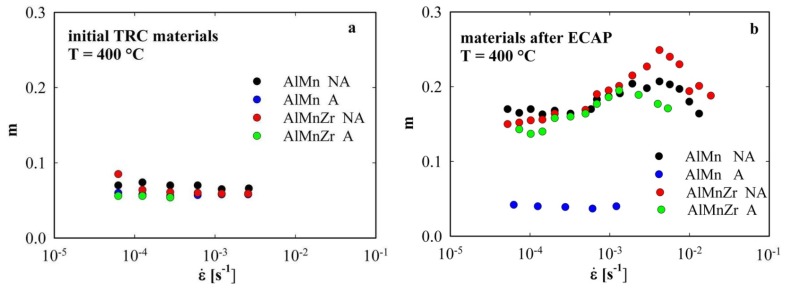
Strain rate sensitivity parameter *m* measured at 400 °C in initial TRC materials (**a**) and materials after four ECAP passes (**b**).

Figure 4 shows the results of the parameter *m* evaluation. All initial TRC materials exhibit very low *m* values much below 0.1 which are independent of the strain rate (a). A similar result was found also in the annealed AlMn alloy after ECAP. On the other hand, much higher *m* values and the marked influence of strain rate on the parameter *m* were observed in the rest of ECAP materials. The maximum value of *m* reaches 0.25 in the non-annealed AlMnZr alloy at the strain rate close to 10^−2^ s^−1^ (b). A good correspondence can be found between the ductility and parameter *m* values (see Table 1). However, it is necessary to take into account the special course of tensile tests (variable strain rate) where ductility does not correspond to a definite strain rate.

**Figure 5:**
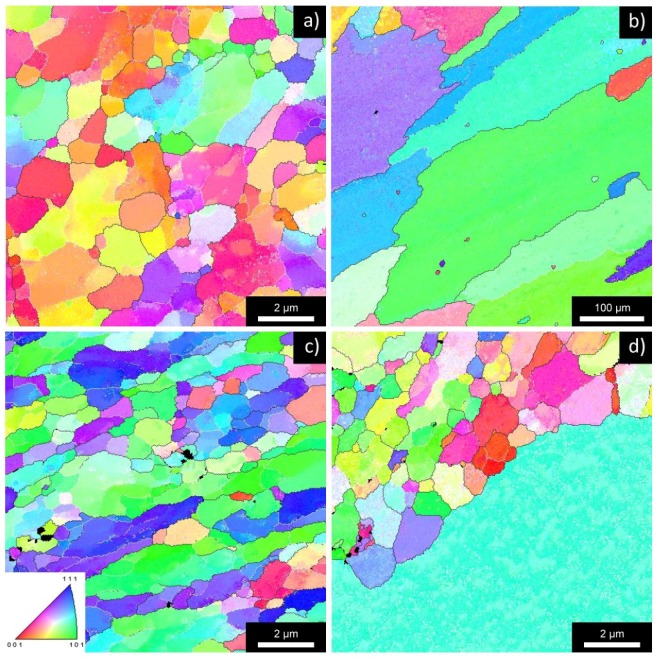
EBSD inverse pole figures of typical areas of the AlMn alloy in the NA state (**a**) and A state (**b**); and of the AlMnZr alloy in the NA state (**c**) and A state (**d**); all ECAP materials after annealing at 400 °C for about 1.5 h.

The differences in mechanical properties reflect the microstructure changes occurring in individual materials at the straining temperature. Figure 5 shows the EBSD maps for typical areas of all studied materials. The investigated area was chosen from the grip regions of tensile samples strained at 400 °C and the observed microstructure documents the influence of pure annealing at this temperature. The total time of exposition to the temperature of 400 °C was about 1.5 h (including the time necessary for heating and temperature stabilization prior to the start of tensile test). Presence of very large grains documents clearly that 400 °C is above the recrystallization temperature for the AlMn alloy in A state (b). This finding is in agreement with the course of the hardness curve of HV for this material and also with low values of ductility and parameter *m*. The microstructure of the AlMnZr alloy in the A state represents a mixture of micrometer grains and a limited number of very large grains (d). Much better structure stability was observed in both alloys in the NA state (a, c). The microstructure is still fine-grained and contains numerous micrometer grains. Statistical processing of EBSD experiments revealed a relatively high fraction of low-angle boundaries with disorientation angles between 5° and 15° (about 50%) in both materials.

**Figure 6:**
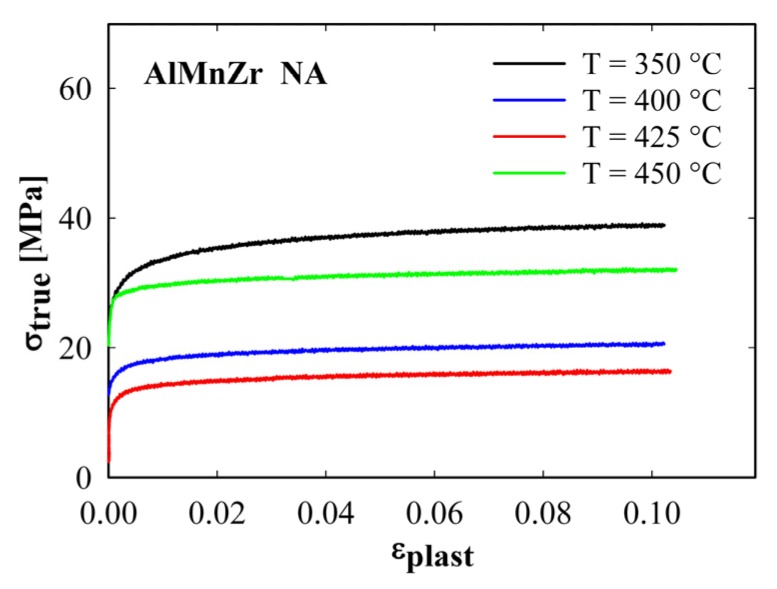
Initial parts of deformation curves of the non-annealed AlMnZr alloy after four ECAP passes at indicated temperatures.

**Figure 7:**
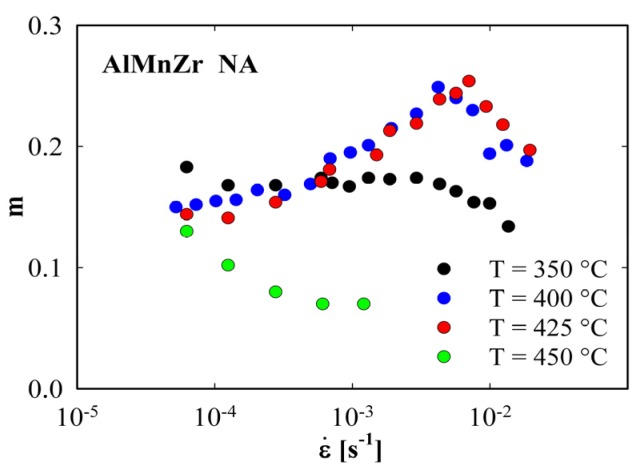
Strain rate dependence of the parameter *m* of the non-annealed AlMnZr alloy after four ECAP passes at indicated temperatures.

The highest values of ductility and parameter *m* were found in the non-annealed AlMnZr alloy after ECAP. This material was therefore selected for the investigation of the influence of straining temperature on the deformation characteristics. The main deformation characteristics found at straining temperatures between 350 and 450 °C are summarized in Table 2. Figure 6 shows the initial parts of deformation curves corresponding to these straining temperatures. The temperature dependence of strength is not monotonously decreasing—a marked increase in strength was observed at the highest temperature of 450 °C. Simultaneously, the ductility was reduced from 119% at 400 °C to 28% at 450 °C. Figure 7 documents the strain rate dependence of the parameter *m*. Whereas the samples strained at 400 and 425 °C exhibit a marked maximum of *m* close to 0.25, further increase in straining temperature to 450 °C results in a drop of *m* values. Again, a good correspondence between the ductility and parameter *m* values was observed.

**Figure 8:**
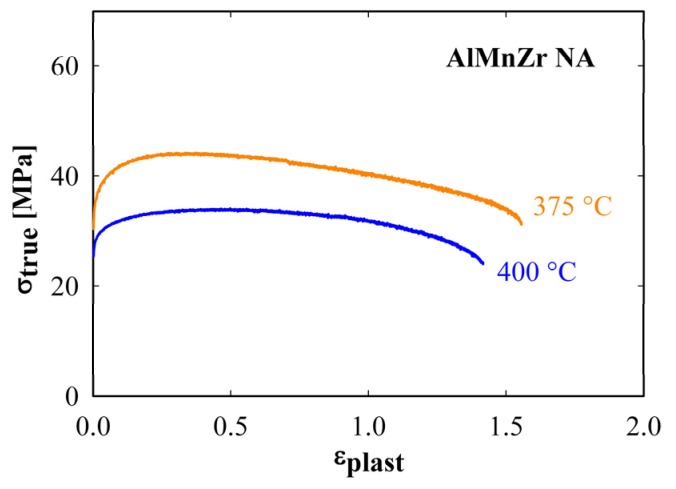
Deformation curve of the non-annealed AlMnZr alloy after four ECAP passes at temperatures 375 and 400 °C and strain rate of 10^−2^ s^−1^.

In order to obtain reliable values of ductility and to verify possible superplastic behavior of the non-annealed AlMnZr alloy the tensile tests at the constant strain rate were carried out. The temperatures of 375 and 400 °C and the strain rate of 10^−2^ s^−1^ were chosen. The temperature of 400 °C corresponds to the maximum of the parameter *m*, the temperature of 375 °C was chosen because of expected better microstructure stability. The deformation curves plotted in Figure 8 reveal the ductility close to 150% at 400 °C and slightly higher at 375 °C. These values are higher than those obtained from special tensile tests with strain rate changes (see Table 1), however, they remain below the bottom limit of superplasticity, which are ductility 150% and *m* = 0.25.

**Table 2:** Deformation characteristics of the non-annealed AlMnZr alloy after four ECAP passes at various temperatures—yield stress (*YS*), ultimate tensile strength (*UTS*), ductility (*A*) and maximum value of the strain rate sensitivity parameter (*m_max_*).

*T* [°C]	*YS* [MPa]	*UTS* [MPa]	*A* [%]	*m_max_*
350	29.2	36.0	97	0.17
400	16.4	19.0	119	0.25
425	12.4	15.2	119	0.25
450	28.2	30.0	28	0.13

**Figure 9:**
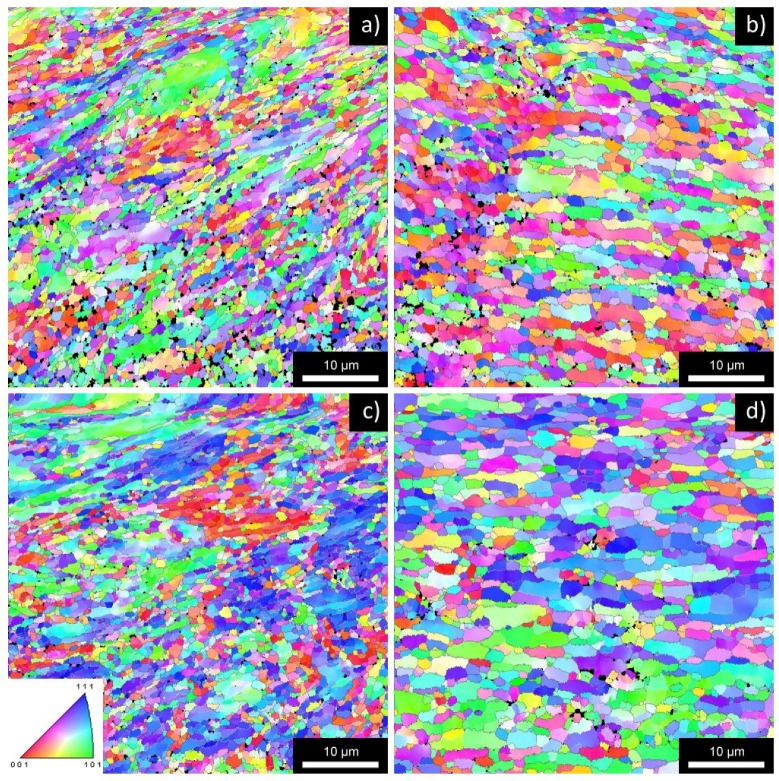
EBSD inverse pole figures of typical areas of the non-annealed AlMnZr after four ECAP passes strained at 375 °C (**a**,**b**) and 400 °C (**c**,**d**); grip regions (**a**,**c**) and regions close to neck (**b**,**d**), tensile axis horizontal.

A detailed analysis of the microstructure was performed using EBSD measurements. Figure 9 brings the EBSD maps of typical areas of both samples strained at 375 and 400 °C. The microstructure of grip regions clearly documents that annealing itself is not able to suppress the directionality typical for ECAP materials. Some areas composed of subgrains were observed at both temperatures. Considering only interfaces with misorientations higher than 15° the mean grain size computed by the linear intercept method is about 1.7 µm for 375 °C and 1.9 µm for 400 °C, respectively. Tensile straining results in a slight grain coarsening in the direction perpendicular to the tensile axis (2.0 µm for 375 °C and 2.2 µm for 400 °C) and especially to a marked elongation of grains along the tensile axis can be seen. The aspect ratio is about 1.6 for both straining temperatures. It should be mentioned that the observed grain elongation of about 60% is significantly lower in comparison with the overall sample elongation of about 150%. The ratio of high-angle boundaries increases from about 40% in the material after ECAP to values slightly above 50% in the only annealed material (grip regions) and to nearly 70% in the strained material close to the neck.

**Figure 10:**
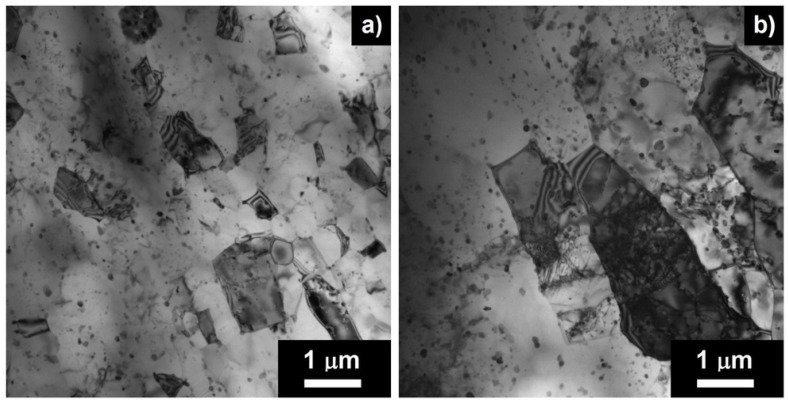
TEM of the non-annealed AlMnZr after four ECAP passes strained at 400 °C, grip region (**a**); region close to the neck (**b**).

The microstructure was additionally investigated using TEM. The micrographs of the non-annealed AlMnZr alloy strained at 400 °C are given in Figure 10. The surviving submicrocrystalline structure was observed in the non-strained grip region, while coarser elongated grains were found close to the neck region.

## 4. Discussion

Similar to other Al-based alloys, ECAP is an effective tool for the microstructure refinement also in TRC Al-Mn-based alloys. The grain size after four ECAP passes is about 400 nm, *i.e.*, slightly lower in comparison with ECAP Al-Zn-based [23] or Al-Mg-based alloys [24]. However, after four ECAP passes the microstructure still contains numerous low-angle boundaries (about 60%) [13]. Similar results were also obtained in the direct chill cast (DC) Al-Mn-Zr-Sc alloy where not fully recrystallized microstructure was observed even after eight ECAP passes [18]. Recovery and recrystallization processes during ECAP are retarded in the studied Al-Mn-based alloys. The explanation can be found in a lower ECAP temperature in the Al-Mn-based alloy in comparison with Al-Zn-based or Al-Mg-based alloys. Despite of the presence of Al_3_Zr particles in the AlMnZr alloy, no dramatic differences in the grain (or subgrain) size were observed neither between ECAP AlMn and AlMnZr alloys nor between annealed A and non-annealed NA materials although the microstructure of the annealed AlMn alloy seems to be coarsest.

Microhardness measurements performed at room temperature confirmed the strengthening effect of ECAP (compare full and open symbols for 20 °C in Figure 2). The introduction of lattice defects and grain refinement occurring during ECAP are believed to contribute significantly to higher hardness. The microhardness prior to isochronal annealing (symbols for temperature 20 °C in Figure 2) is increased by the addition of Zr both in the initial TRC (open symbols) and in the ECAP materials (full symbols) probably due to the presence of Al_3_Zr particles. Nevertheless, the long term annealing at 450 °C prior to ECAP which should promote precipitation of Al_3_Zr and α-Al_15_(Mn,Fe)_3_Si_2_ particles results unexpectably in a lower hardness of both ECAP materials. This observation can be explained assuming that Mn atoms dissolved in Al-matrix are the most important structural features decelerating the process of dynamic recovery and recrystallization during ECAP. The precipitation of the α-Al_15_(Mn,Fe)_3_Si_2_ during annealing is accompanied by a depletion of Al-matrix by Mn atoms and the process of dynamic recovery and recrystallization is thus faster in the annealed material and the resulting microhardness is lower.

The phase composition influences the development of microstructure and mechanical properties during exposition of all ECAP materials to elevated temperatures as documented by hardness curves in Figure 2. The slow decrease of microhardness starting already at 150 °C in all materials can be interpreted as a recovery of dislocation substructure whereas the final steep decrease can be attributed to recrystallization and grain growth [25]. The lowest stability observed in the annealed AlMn alloy can be explained by the absence of Al_3_Zr particles and by a depletion of matrix by Mn atoms.

The temperature of 400 °C selected for high temperature tensile tests corresponds to the end of recrystallization stage in the annealed AlMn and to the beginning of this stage for other materials. It can be expected that deformation characteristics measured at this temperature will reflect the ability of individual materials to retain their fine-grained structure. A general feature documented in Table 1 and Figure 3 is lower high temperature strength of ECAP materials in comparison with much coarser grained initial TRC materials. Such softening due to grain size decrease is typical for superplastic materials where grain boundaries play a decisive role in the deformation process [26].

Structural superplasticity is a high temperature phenomenon and the most of superplastic Al-based alloys exhibit this behavior at temperatures between 400 and 500 °C. The enormous grain refinement to the submicrocrystalline range due to ECAP can displace the region of superplastic behavior either to high strain rates (high strain rate superplasticity [27]) or to lower straining temperatures (low temperature superplasticity [28]). The stability of very fine-grained microstructure is a necessary condition for this behavior. This requirement is evidently not fully fulfilled in our Al-Mn-based materials.

The EBSD experiments show that full recrystallization and a vast grain growth occurred in the annealed AlMn alloy at 400 °C where these restoration processes are not retarded by Al_3_Zr particles and by a sufficient concentration of Mn atoms dissolved in the matrix. This microstructure development is in agreement with the hardness curve and also with low values of ductility and strain rate sensitivity. The deformation mechanism of this material is based predominantly on the motion of lattice dislocations through the matrix with numerous Mn, Fe, and Si containing particles. The observed quasi-brittle fracture inclined about 45° to the tensile axis suggests that deformation is localized in shear bands.

The annealed AlMnZr alloy exhibits a mixed microstructure composed of large coarsened grains and surviving fine grains due to the presence of Al_3_Zr particles that can partially retard the grain growth. Therefore the deformation mechanism is a mixture of processes occurring in coarse grained regions (the same as mentioned above for the annealed AlMn alloy) and processes occurring in fine-grained regions. The role of boundaries as sites for annihilation of lattice dislocation and as carriers of plastic deformation through grain boundary sliding has to be considered. Moderate values of ductility (61%), parameter *m* below 0.2, and quasi-brittle fracture in a shear band suggests the dominance of a dislocation mechanism.

Both non-annealed alloys retain their fine-grained structure during exposition to 400 °C for the time of about 1.5 h which is necessary for the complete tensile test. This is in agreement with results of isothermal annealing [11]. The deformation behavior reveals some indications of superplastic deformation—an increase in ductility to values above 100%, an increase of parameter *m* to 0.25, and especially the appearance of strain rate dependence of the parameter *m* with a characteristic maximum. The elongation of individual grains is significantly lower than the overall sample elongation. All these indications suggest the role of grain boundaries in the deformation process. The strain is more homogeneously distributed within the tensile sample and the observed fracture occurs by the diffuse necking mode. The grain coarsening in the strained parts of tensile samples is connected with elimination of low-angle boundaries and may be, at least partially, explained by the process of subgrain coalescence.

The experiments performed on the non-annealed AlMnZr alloy at various temperatures confirm previously mentioned conclusions. The “superplastic like” behavior is observed only in a very narrow temperature range close to 400 °C where the fine-grained microstructure is still retained. The expected instantaneous grain growth at 450 °C results in a decrease of ductility and parameter *m*. The grain coarsening is also responsible for the stress increase at higher straining temperatures.

The ductility and parameter *m* values measured at 400 °C are within the experimental error identical with those found in the ECAP Al-Mn-Zr-Sc alloy prepared by direct chill casting [18]. A principal difference between the TRC material without Sc and conventional materials with Sc is observed at higher deformation temperatures. A higher content of Zr (0.23 wt.%) and addition of Sc (0.27 wt.%) result in a significant enhancement of retarding forces for grain boundary migration, preservation of fine-grained microstructure, and retaining of “superplastic like” deformation behavior up to 500 °C in the Al-Mn-Zr-Sc alloy [18].

## 5. Conclusions

(1) Very fine-grained microstructure (*d* ~ 400 nm) can be produced in the TRC Al-Mn-based alloys using ECAP.
(2) Recrystallization and vast grain growth occur at temperatures between 350 and 450 °C. Addition of Zr enhances the microstructure stability. Thermal treatment at 450 °C prior to ECAP has an opposite effect as it reduces the content of Mn atoms dissolved in the matrix enhancing thus the recovery and recrystallization processes.
(3) The most stable non-annealed AlMnZr alloy exhibits the highest ductility and strain rate sensitivity parameter *m* values at 400 °C. These values are below the bottom limits of superplasticity (ductility 150%, *m* = 0.25), but much higher than those found in coarse grained materials.
(4) The “superplastic like” behavior, especially the strain rate dependence of the parameter *m* and the increase of stress with increasing grain size suggest that grain boundaries play a significant role in the deformation mechanism.
